# Low vector competence in sylvatic mosquitoes limits Zika virus to initiate an enzootic cycle in South America

**DOI:** 10.1038/s41598-019-56669-4

**Published:** 2019-12-27

**Authors:** Rosilainy S. Fernandes, Maria I. Bersot, Marcia G. Castro, Erich Loza Telleria, Anielly Ferreira-de-Brito, Lidiane M. Raphael, Myrna C. Bonaldo, Ricardo Lourenço-de-Oliveira

**Affiliations:** 1 Laboratório de Mosquitos Transmissores de Hematozoários. Instituto Oswaldo Cruz - FIOCRUZ, Rio de Janeiro, Brazil; 2 Laboratório de Biologia Molecular de Parasitas e Vetores, Instituto Oswaldo Cruz – FIOCRUZ, Rio de Janeiro, Brazil; 3 Laboratório de Biologia Molecular de Flavivírus. Instituto Oswaldo Cruz - FIOCRUZ, Rio de Janeiro, Brazil; 4 Department of Parasitology, Faculty of Science, Charles University, Vinicna 7, 128 44, Prague, 2 Czech Republic

**Keywords:** Parasite host response, Viral vectors, Infectious-disease diagnostics

## Abstract

Zika virus (ZIKV) has spread in the Americas since 2015 and the potential establishment of a sylvatic transmission cycle in the continent has been hypothesized. We evaluated vector competence of five sylvatic Neotropical mosquito species to two ZIKV isolates. Distinct batches of *Haemagogus leucoceleanus*, *Sabethes albiprivus*, *Sabethes identicus*, *Aedes terrens* and *Aedes scapularis* females were respectively orally challenged and inoculated intrathoracically with ZIKV. Orally challenged mosquitoes were refractory or exhibited low infection rates. Viral dissemination was detected only in *Hg. leucocelaenus*, but with very low rates. Virus was not detected in saliva of any mosquito orally challenged with ZIKV, regardless of viral isolate and incubation time. When intrathoracically injected, ZIKV disseminated in high rates in *Hg. leucocelaenus*, *Sa. identicus* and *Sa. albpiprivus*, but low transmission was detected in these species; very low dissemination and no transmission was detected in *Ae. terrens* and *Ae. scapularis*. Together these results suggest that genetically determined tissue barriers, especially in the midgut, play a vital role in inhibiting ZIKV for transmission in the tested sylvatic mosquito species. Thus, an independent enzootic transmission cycle for ZIKV in South America is very unlikely.

## Introduction

Zika virus (ZIKV) (*Flaviviridae*, genus *Flavivirus*) was discovered in the Zika forest in Uganda, Africa, in 1947^1^. Only sporadic human infections of ZIKV were documented in the following decades in Africa and Asia^2^. The virus has diverged into two main genetic lineages: African and Asian^3^. In 2007, a large outbreak of the Asian genotype erupted on Yap Island^4^, and between 2013 and 2014 the virus reached French Polynesia and New Caledonia, infecting a large fraction of the population of these islands^5^. After the first cases were recorded in Northeast Brazil in early 2015^6,7^, ZIKV spread rapidly throughout tropical and subtropical areas in the Americas and caused an unprecedented epidemic. In 2016, 211,770 probable cases of ZIKV were recorded in Brazil (incidence rate of 103.6 cases/100 thousand inhabitants) (https://bit.ly/2xIOcta). The seriousness of the problem increased with the association of ZIKV infections with congenital troubles such as microcephaly^8–11^. From 2015 to 2019, autochthonous transmission of ZIKV has been reported in 52 American countries and territories and the continent has recorded 829,944 and 246,038 probable and confirmed cases, respectively (http://www.paho.org/data/index.php/en/mnu-topics/zika.html).

ZIKV is an arbovirus transmitted primarily in two ecological cycles. Urban epidemic transmission of ZIVK is vectored by the domestic and anthropophilic mosquitos *Aedes* (*Stegomyia*) *aegypti*^3^. The enzootic cycle has been documented to occur only in Africa, where the virus circulates between non-human primates (NHPs) and sylvatic mosquitoes with humans typically accidental hosts during epizootics^3,12^. *Aedes* (*Stegomyia*) *africanus* was the first mosquito incriminated as a sylvatic ZIKV vector in Uganda^1^. Other *Aedes* species, especially those belonging to the subgenus *Stegomyia* (*e.g. Ae. aegypti, Ae. furcifer, Ae. luteocephalus, Ae. taylori, Ae. vittatus*), have been confirmed or suspected as ZIKV vectors in sylvatic and partially human modified environments in Africa^3,13^. In Africa, these *Aedes* mosquitoes are coincidently the vector of another *Flavivirus*, the yellow fever virus (YFV). Yellow fever has probably spread from Africa to the Americas during the African slave trade^14^, where YFV was probably transmitted initially in urban sites, and subsequently initiated a sylvatic transmission cycle in tropical and subtropical American zones following the virus’ adaptation to Neotropical NHPs and arboreal primatophilic mosquitoes of genera *Haemagogus* and *Sabethes*^15–17^).

Epidemiological history of YFV well illustrates how an arbovirus originally circulating among wild animals may spillover transmission to humans in the modified environment, and then to re-establish a wild transmission cycle again (spillback). This likely occurs when viremic humans infected in the urban cycle enter forest areas potentially initiating a sylvatic cycle if local mosquitoes are competent to transmit the virus and there are susceptible amplification hosts^18^. Since entering South America, ZIKV has infected thousands of people living in the proximity to forest areas and it is highly likely viremic individuals have been bitten by sylvatic mosquitoes. An enzootic cycle makes eradication of virus transmission much more difficult as exemplified by the case of YFV in Brazil^16^.

In this study, we tested whether sylvatic Neotropical mosquito species belonging to the genera *Haemagogu*s, *Sabethes* and *Aedes* are experimentally competent to transmit ZIKV isolates of the Asian genotype isolated from Brazil.

## Methodology

### Ethics statements

This study was approved by the Institutional Ethics Committee on Animal Use (CEUA-IOC license LW-34/14) at the Instituto Oswaldo Cruz. Mosquito collections in the Atlantic forest in Rio de Janeiro were approved by local environmental authorities (PNMNI license 001/14-15; SISBIO-MMA licenses 37362-2 and 012/2016). This study did not involve endangered or protected species.

### Mosquitoes

We used female mosquitoes of five species of wild mosquitoes from Brazil that are wide spread in South America: *Haemagogus leucocelaenus, Aedes terrens, Aedes scapularis, Sabethes identicus* and *Sabethes albiprivus*. These three first species have never been successfully colonized in laboratory^19,20^. Thus, tested females of *Hg. leucocelaenus* and *Ae. terrens* were directly derived from field-collected eggs with ovitraps in Parque Natural Municipal de Nova Iguaçu (22°46′45″S 43°27′23″W) as previously described^20^, while those of *Ae. scapularis* resulted from eggs laid in the laboratory by gravid wild females captured in Manguinhos (22°52′20″S 43°14′46″W). Eggs of these three species were hatched by immersion in dechlorinated tap water for two consecutive days. In the cases of *Sa. albiprivus and Sa. identicus*, we used females from colonies established in the laboratory since 2013 from specimens collected in Reserva Biológica do Tinguá (22° 32′ 43″ S, 43° 23′ 5″ W)^17^. Larvae were reared in pans (~50 larvae/pan measuring 25 × 25 × 10 cm) containing 1 liter of dechlorinated tap water, supplemented with yeast powder and shed leaves (for the non-colonized Aedini species), renewed every 2–3 days. Adults were kept in 30 × 30 × 30-cm mesh cages, maintained in an insectary (28 ± 1 °C; 80 ± 10% RH; 12 h:12 h light:dark cycle) and supplied with honey solutions.

### Virus

Mosquitoes were challenged with two ZIKV isolates of the Asian genotype, named Rio-U1 and Rio-S1, respectively obtained by Bonaldo *et al*.^21^ from urine and saliva of two patients in January 2016, living in distinct districts in Rio de Janeiro. Viral stocks were obtained after two passages of the isolates onto Vero cells and kept at −80 °C until use. The comparison of genomic sequences of these ZIKV isolates [Rio-U1 (KU926309); Rio-S1 (KU92630)] yielded 99.6% identity, displaying six amino acid variations in the viral proteins.

### Oral challenge assays

To access vector competence, we isolated lots of 40–50 female mosquitoes with 5–7day-old in feeding boxes and starved 48 h before orally challenge with ZIKV. The infectious meal consisted of 2 mL of a mixture of two parts of washed rabbit erythrocytes and one part of the viral suspension containing a final viral titer of 10^6^ PFU/mL with a phagostimulant (0.5 mM ATP). Females were fed through a pig-gut membrane covering the base of glass feeders containing the infectious blood-meal maintained at 37 °C. Mosquito feeding was limited to 1 h. Only fully engorged females were incubated at 26 °C constant temperature, 70 ± 10% RH and 12 h:12 h light: dark cycle, with daily access to honey solution. As expected for sylvatic mosquitoes, the artificial blood-feeding rates under experimental conditions here were low (<15%), especially for *Sa identicus* (1%). When available, samples of 30 mosquitoes of each species were examined at 7, 14 and 21 days after virus exposure, abbreviated as “dpi”.

### Intrathoracic Injection

Female mosquitoes were briefly anesthetized with ice and individually maintained on a glass plate over ice during injection. Insects were gently handled with forceps and injected with a pulled glass capillary and handheld microinjector (Nanoject II, Drummond Sci.) under a stereomicroscope. Each individual was injected into its thorax (pleural membrane) with 32.2 *n*L of ZIKV Rio-S1 stock containing 10^6.5^ PFU/mL and subsequently incubated at the same conditions above described for 10 days until examination.

### Mosquito analyses

Mosquitoes were individually processed as follows: abdomen and thorax (herein after referred to as body) were analyzed to estimate viral infection rate, head for dissemination and saliva for transmission. One female was handled at a time, by using disposable and disinfected supplies to avoid contamination between individuals and between tissues of the same mosquito as previously described^22^. For the determination of viral infection and dissemination rates, each mosquito body and head were respectively ground in 500 μL and 250 μL of Earle’s 199 medium supplemented with 4% FBS, and centrifuged at 10,000 × *g* for 5 min at 4 °C before titration.

### Plaque assays

Body and head homogenates were serially diluted and inoculated onto monolayers of Vero cells in 96-well plates. After 1 h incubation of homogenates at 37 °C, 150 μL of 2.4% CMC (carboxymethyl cellulose) in Earle’s 199 medium was added per well. After 7 days incubation at 37 °C, cells were fixed with 10% formaldehyde, washed, and stained with 0.04% crystal violet. Presence of viral particles was assessed by detection of viral plaques (plaque forming unit, PFU).

### RT-qPCR

In order to check the results from plaque assays in Vero cells, body and head homogenates were individually submitted to specific ZIKV RNA detection and quantification through RT-qPCR, using the SuperScript III Platinum one-step RT- qPCR (Invitrogen) in QuantStudio 6 Flex Real-Time PCR System (Applied Biosystems). For each reaction, we used 600 nM forward primer (5′-CTTGGAGTGCTTGTGATT-3′, genome position 3451–3468), 600 nM reverse primer (5′-CTCCTCCAGTGTTCATTT-3′, genome position 3637–3620) and 800 nM probe (5′FAM- AGAAGAGAATGACCACAAAGATCA-3′TAMRA, genome position 3494–3517) as previously described. The reverse transcription was performed at 45 °C for 15 min. The qPCR conditions were 95 °C for 2 minutes, followed by 40 amplification cycles of 95 °C for 15 sec, 58 °C for 5 sec and 60 °C for 30 sec. For each run, numbers of ZIKV RNA copies were calculated by absolute quantitation using a standard curve, whose construction details are described elsewhere^21^.

With the intention of assessing the transmission rate (TR), mosquito saliva was collected in individual pipette tips containing 5 μL FBS and processed by PFU assays, as previously described^23^. Accordingly, mosquito saliva was inoculated onto Vero Cell monolayer in 6-well plates subsequently incubated for 7 days at 37 °C, under 3 mL of 2.4% CMC in Earle’s 199 medium overlay and stained as described above. Viral titers of saliva were expressed as PFU/saliva. Only saliva from mosquitoes which presented dissemination were analyzed.

Infection rate (IR) was determined only for orally challenged mosquitos and refers to the proportion of mosquitoes with infected body (abdomen and thorax) among engorged individuals. Disseminated infection rate (DIR) corresponds to the proportion of mosquitoes with infected head among infected mosquitoes (i.e.; abdomen/thorax positive) whether after intrathoracic injection or orally challenged with virus. Transmission rate (TR) represents the proportion of mosquitoes with infectious saliva among mosquitoes with disseminated infection. Confidence intervals of 95% were calculated for all rates and combination of mosquito species, virus isolate and incubation time. The Wilcoxon signed rank test was used to compare the viral load in saliva. Data analyses were done with PRISM 5.0 software (GraphPad Software, San Diego-CA, USA, 2007).

## Result

**Figure 1:**
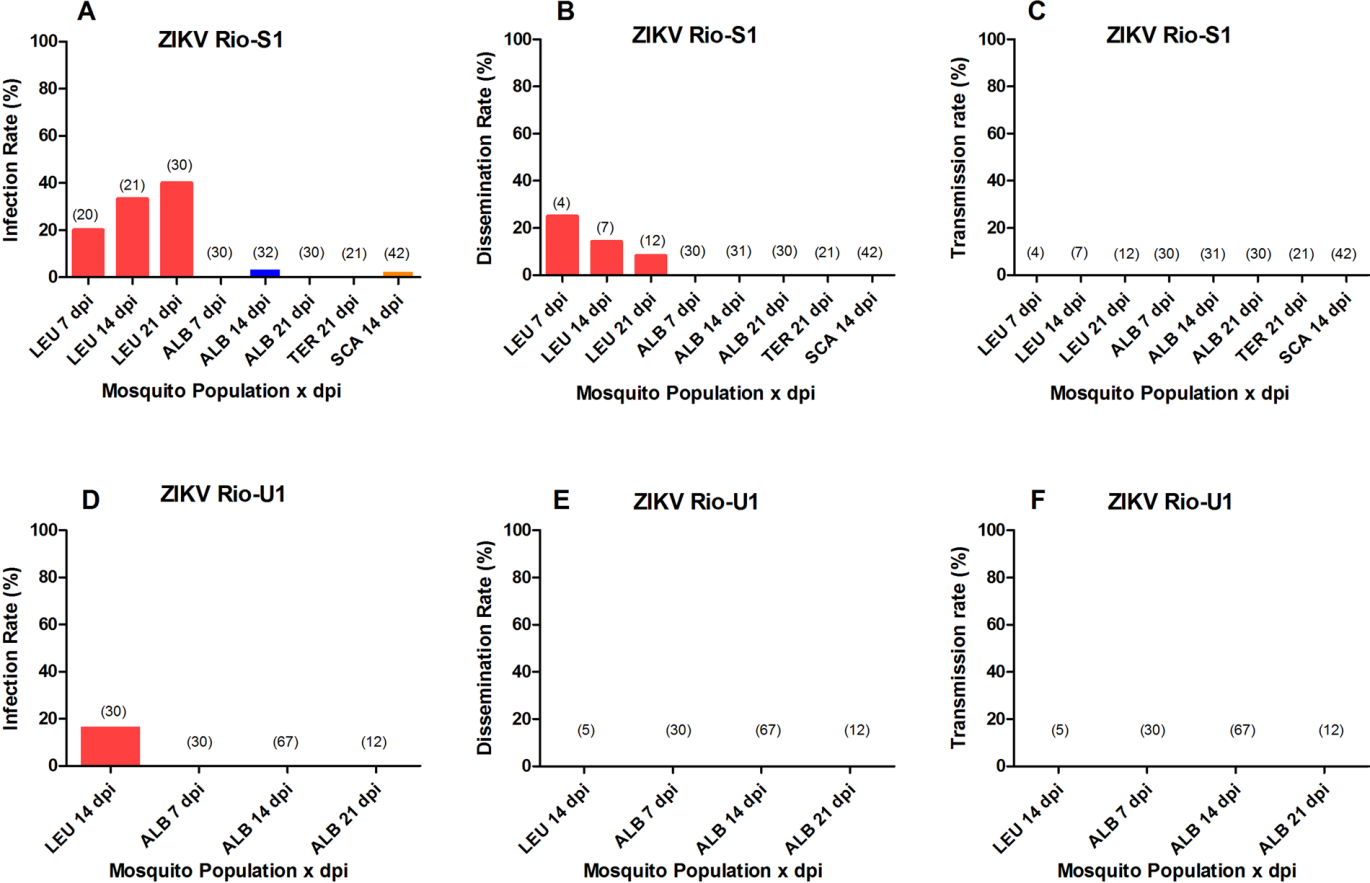
Viral infection (**A,D**), dissemination (**B,E**), transmission (**C,F**) rates at days 7, 14 and 21 after oral challenging of *Hg leucocelaenus* (LEU), *Sa albiprivus* (ALB), *Aedes terrens* (TER), *Ae scapularis* (SCA) and *Sa.identicus* females respectively with two Zika virus isolates - Rio-S1 and Rio-U1. The number of mosquitoes analyzed is in parentheses on top of bars.

We assessed vector competence of five sylvatic Neotropical mosquito species by orally challenging females with two ZIKV isolates. Overall, almost all were refractory to infection or exhibited low IR (Fig. 1A,D; Table S1). None of the challenged *Ae. terrens, Ae. scapularis* and *Sa. identicus* mosquitoes became infected or could sustain ZIKV replication in the midgut. Infection was detected in a single *Sa. albiprivus* examined at 14 dpi out of the total of 201 challenged with the two ZIKV isolates regardless the incubation time (0.5%). Infection rates in *Hg. leucocelaenus* were also low, ranging from 14.8% (ZIKV Rio-U1, 14 dpi) to 40% (ZIKV Rio-S1, 21 dpi) of engorged females and increased with incubation time.

Viral dissemination was not detected in any tested species except in *Hg. leucocelaenus* challenged with the ZIKV Rio-S1 isolate, where only from 3.3% (21 dpi) to 5% (7 and 14 dpi) of individuals initially taking the blood infectious meal had virus in head homogenates. When considering only those *Hg. leucocelaenus* females with infected body, maximum DIR value was 25% at 7 dpi and decreased with incubation time (Fig. 1B,E; Table S1). We assumed that ZIKV detected in the assayed *Hg. leucocelaenus* were result of replication of the virus isolate we used, and not acquired via vertical transmission as 100 *Hg. leucocelaenus* adult females randomly selected among the ones emerged from the same area and egg batch of those we challenged were negative when screened by Rt-qPCR. Transmission (virus present in saliva) was not detected in any mosquito orally challenged with ZIKV, regardless of viral isolate and incubation time.

**Figure 2:**
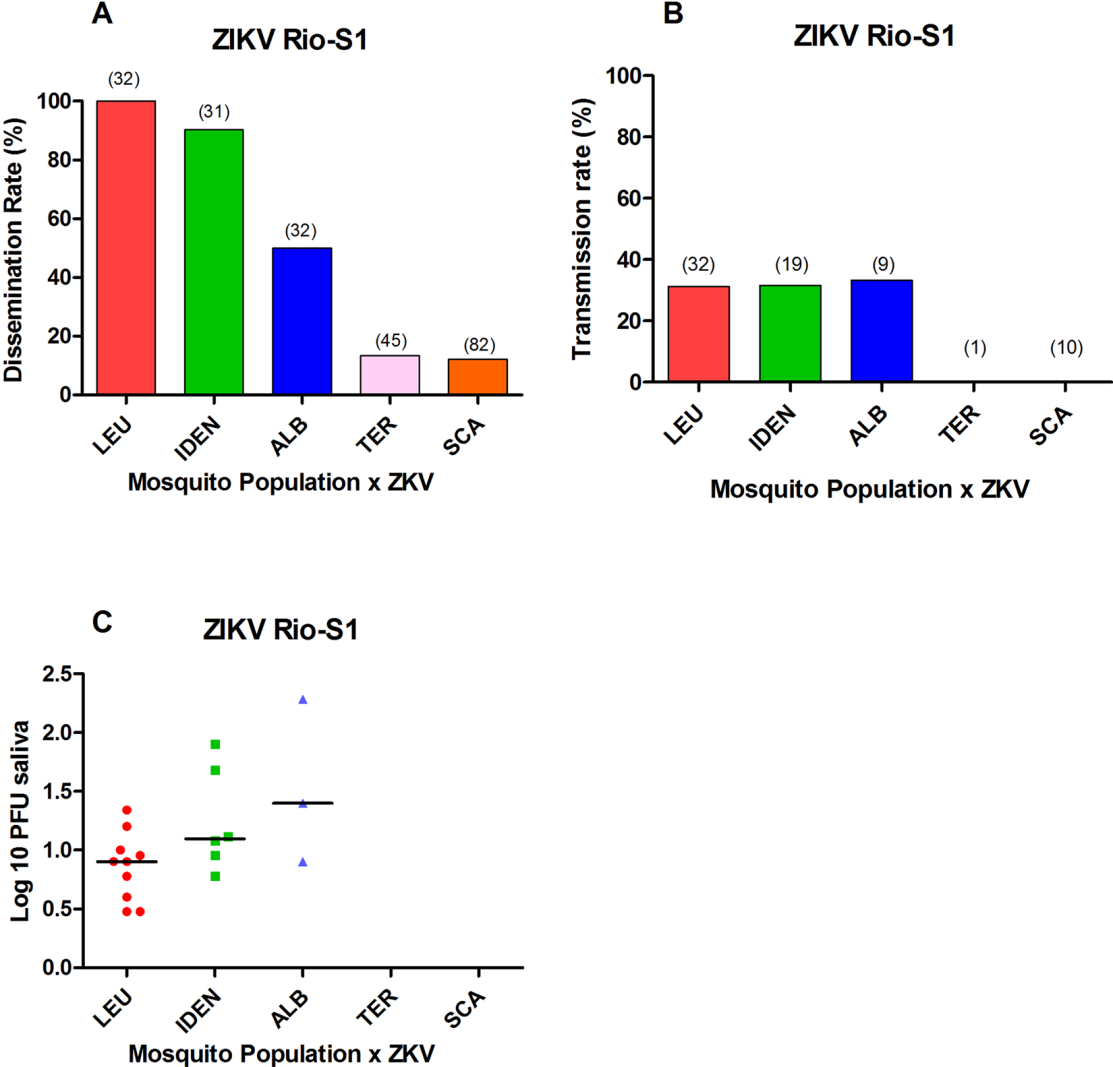
Viral dissemination (**A**), transmission (**B**) rates and viral load (**C**) in saliva of *Hg. leucoceleanus* (LEU), *Sa. identicus* (IDEN), *Sa. albiprivus* (ALB) *Ae. terrens* (TER) and *Ae. scapularis* (SCA) at day 10 after intrathoracic injection of Zika virus (Rio-S1 isolate). Viral load was determined by plaque assays in Vero cells. The number of mosquitoes analyzed is in parentheses on top of bars.

To test if bypassing mosquito midgut infection barrier (MIB) would help ZIKV dissemination and transmission in the same sylvatic mosquito species we conducted intrathoracic inoculation of viral particles into the haemocoel. Dissemination (defined by presence of virus in the head) of ZIKV was attained in all five species, however at very contrasting rates (Fig. 2A, Table S1). All injected *Hg. leucocelaenus* sustained ZIKV replication in head tissues, but transmission was achieved in only 31.2%. An essentially similar behavior was recorded for both injected *Sabethes* species. Viral load in saliva did not differ (p = 0.4203) between intrathoracically injected *Hg. leucoceleanus*, *Sa. identicus* and *Sa. albiprivus*. On the other hand, ZIKV disseminated in only 12.1% and 13.3% of injected *Ae. scapularis* and *Ae. terrens*, which did not expectorate ZIKV infected saliva (Fig. 2; Table S1).

## Discussion

Since ZIKV spread in the Americas in 2015, the possibility of establishment of a sylvatic transmission cycle has been raised^24^. Risk assessment of this spillover has taken into consideration results from mathematical dynamic transmission models, the search of evidences of ZIKV natural infections especially in NHPs and evaluations of vertebrate host competence to mount sustain viremia sufficient to infect a mosquito^25–29^. Here, we evaluated vector competence for ZIKV transmission of five sylvatic Neotropical mosquito species widely distributed in South America. We tested two ZIKV isolates belonging to the genotype circulating in the Americas, crucial for a realistic risk assessment^24^. Vector competence consists of the ability of a mosquito to become orally infected and disseminate (escape midgut) and transmit (infect saliva) the virus through the bite upon subsequent feedings. Our results showed that orally challenged *Ae. terrens, Ae. scapularis, Sa. identicus*, *Sa. albiprivus* and *Hg. leucocelaenus* mosquitoes from Brazil were totally refractory or only very low infection rates were noticed; dissemination was detected only in *Hg. leucocelaenus* at a very low rate. Importantly, no transmission was detected regardless of the orally challenged mosquito species, ZIKV isolate, and incubation period considered. These results agree with the very low vector competence exhibited by *Sabethes cyaneus* of a colony that originated from Panama and were orally challenged with a Mexican ZIKV strain^30^.

The low to null IRs and DIRs displayed by orally challenged sylvatic Brazilian mosquito species is in strong contrast to the high rates detected for several Brazilian *Ae. aegypti* populations that we have previously challenged with the same ZIKV isolates and viral titers in the blood meal^23,31^. Interestingly, in contrast to the refractoriness or low susceptibility to ZIKV, *Ae. terrens* and *Hg. leucocelaenus*, as well as *Sa. albiprivus* and *Hg. leucocelaenus* from the same localities were respectively competent to transmit chikungunya virus^20^ and YFV^17^ in the laboratory. Taken together, these data strengthen the evidence of important genetically determined tissue barriers to infection with ZIKV in Brazilian sylvatic mosquitoes.

The two preliminary genetically determined barriers to viral transmission are the MIB, that prevents invasion and replication of the viruses, and the midgut escape barrier (MEB) that prevents dissemination to other tissues^32,33^. When bypassing the MIB by intrathoracic inoculation of ZIKV in sets of the same sylvatic mosquito species, ZIKV intensely disseminated into the head and transmission was achieved in *Hg. leucocelaenus*, *Sa. identicus* and *Sa. albpiprivus*, while low dissemination and null transmission was noticed in *Ae. terrens* and *Ae. scapularis*. These results suggest a strong role for midgut barriers, as well as salivary gland barriers, in selecting ZIKV for infection and transmission in these sylvatic species.

Interestingly, although high rates of intrathoracically injected *Sabethes* and *Haemagogus* sustained ZIKV replication, transmission was distinctly reduced in these genera and not detected in the two *Aedes* species tested, suggesting an additional role of salivary glands as a barrier to ZIKV transmission. It also may indicate that the tested Neotropical *Aedes* have different barriers for selecting ZIKV transmission than *Ae. aegypti* and congeneric African species involved in the sylvatic cycle in the Old World.

Brazil and other South American countries present a favorable environment for the establishment and spread of arboviruses, as amply illustrated by historical epidemiological data for arboviruses transmitted by invasive *Ae. aegypti*^25,34^. However, the establishment of a sylvatic transmission cycle of ZIKV is likely dependent on several factors, among which are the existence of competent forest primatophilic mosquito species, which intersect repeatedly with susceptible and vertebrate amplification hosts in a favorable environment^18^. From the point of view of vertebrate hosts, some neotropical NHP species have been shown to be susceptible to ZIKV infection in the laboratory and the detection by PCR of ZIKV genomic fragments in marmosets and capuchin monkeys captured in urban/pariurban ZIKV epidemic/endemic areas has been reported^27–29^. But, no viremic and immune forest-caught NHPs have yet been found. There is still no evidence of an independent ZIKV sylvatic transmission cycle (NHP-sylvatic mosquito-NHP) in South America. Perhaps, refractoriness and very low vector competence of sylvatic mosquitoes described here and elsewhere^30^ block the initiation of such enzootic cycles in this continent. However, repeated feeding on viremic vertebrate hosts with recurrent interactions with virus may lead to adaption of the virus to be transmitted by sylvatic mosquitoes^35^. Thus, continued surveillance of ZIKV circulation in animals and mosquitoes should be implemented in Brazil and other South American countries due to their great diversity and abundance of sylvatic primatophilic acrodendrophilic mosquito species. The establishment of a sylvatic transmission cycle of ZIKV in the Americas would make control efforts very difficult and eradication likely impossible.

## Conclusion

*Hg. leucocelaenus*, *Sa. identicus*, *Sa. albpiprivus*, *Ae. terrens* and *Ae. scapularis*, sylvatic mosquito species widely spread in South America, are refractory to ZIKV or exhibit low vector competence, a decidedly limiting factor for the virus to initiate an enzootic independent transmission cycle in the continent.

## Supplementary information

Supplementary information 
