# Author Correction: Low vector competence in sylvatic mosquitoes limits Zika virus to initiate an enzootic cycle in South America

**DOI:** 10.1038/s41598-021-91676-4

**Published:** 2021-06-10

**Authors:** Rosilainy S. Fernandes, Maria I. Bersot, Marcia G. Castro, Erich Loza Telleria, Anielly Ferreira-de-Brito, Lidiane M. Raphael, Myrna C. Bonaldo, Ricardo Lourenço-de-Oliveira

**Affiliations:** 1 Laboratório de Mosquitos Transmissores de Hematozoários, Instituto Oswaldo Cruz - FIOCRUZ, Rio de Janeiro, Brazil; 2 Laboratório de Biologia Molecular de Parasitas e Vetores, Instituto Oswaldo Cruz – FIOCRUZ, Rio de Janeiro, Brazil; 3 Laboratório de Biologia Molecular de Flavivírus, Instituto Oswaldo Cruz - FIOCRUZ, Rio de Janeiro, Brazil; 4 Department of Parasitology, Faculty of Science, Charles University, Vinicna 7, 128 44 Prague, 2, Czech Republic

Correction to: *Scientific Reports* https://doi.org/10.1038/s41598-019-56669-4, published online 27 December 2019

In the original version of this Article the Funding section was omitted.

The Funding section now reads:

“Fundação de Amparo a Pesquisa do Estado do Rio de Janeiro FAPERJ (grants E-26/102.351/2013, E-26/201.335/2016, E-26/202.431/2019), CNPq (grant 309577/2013-6), and European Union's Horizon 2020 Research and Innovation Programme under ZIKAlliance (grant Agreement no. 734548).”

The original Article has been corrected.

