# Supplementary material for: Low vector competence in sylvatic mosquitoes limits Zika virus to initiate an enzootic cycle in South America
Source: Sci Rep. 2019 Dec 27;9:20151. doi: 10.1038/s41598-019-56669-4 (PMC6934573; doi:10.1038/s41598-019-56669-4)
Supplement: Supplementary file 1 — Supplementary information [file 41598_2019_56669_MOESM1_ESM.pdf]

Low vector competence in sylvatic mosquitoes limits Zika virus to initiate an enzootic cycle in South America

Fernandes RS[1], Bersot, MI[1], Castro MG[1], Erich Loza Telleria[2,4], Ferreira-de-Brito A[1], Raphael L[3], Bonaldo, MC[3], Lourenço-de-Oliveira, R[1*]

[1] Laboratório de Mosquitos Transmissores de Hematozoários. Instituto Oswaldo Cruz - FIOCRUZ, Rio de Janeiro, Brazil .

[2] Laboratório de Biologia Molecular de Parasitas e Vetores, Instituto Oswaldo Cruz – FIOCRUZ, Rio de Janeiro, Brazil.

[3] Laboratório de Biologia Molecular de Flavivírus. Instituto Oswaldo Cruz - FIOCRUZ, Rio de Janeiro, Brazil.

[4] Department of Parasitology, Faculty of Science, Charles University, Vinicna 7, 128 44, Prague 2, Czech Republic.

*Correspondence to [lourenco@ioc.fiocruz.br].

Supplementary Table 1

Infection, dissemination and transmission rates of wild mosquitoes from Brazil challenged with two Zika virus isolates

**Mosquito Species**	**Virus isolate**	**dpi**	**Number Engorged survivors**	**Infection Rate**			**Dissemination Rate**			**Transmission Rate**		
**Oral challenge**				N[a] positive/ engorged	IR%	95% CI[e]	N[b] positive/ infected	DIR%	95% CI[e]	N[c] positive / disseminated	TR%	95% CI[e]
Hg. leucocelaenus	ZIKVRio-S1	7<br>14<br>21	20<br>21<br>30	4/20<br>7/21<br>12/30	20<br>33.3<br>40	- 9.5 : 9.5<br>15.9 : 49.2<br>- 19.1 : 59.1	1/4<br>1/7<br>1/12	25<br>14.2<br>8.3	- 11.9 : 36.9<br>- 6.8 : 21<br>- 3.9 : 12.2	0/1<br>0/1<br>0/1	0<br>0<br>0	-<br>-<br>-
Hg. leucocelaenus	ZIKVRio-U1	14	30	4/30	14.8[d]	--	0/0	0	--	0/0	--	--
Sa. albiprivus	ZIKVRio-U1	7<br>14<br>21	30<br>67<br>12	0<br>0<br>0	0<br>0<br>0	--<br>--<br>--	0/0<br>0/0<br>0/0	0<br>0<br>0	--<br>--<br>--	0/0<br>0/0<br>0/0	--<br>--<br>--	--<br>--<br>--
Sa. albiprivus	ZIKVRio-S1	7<br>14<br>21	30<br>32<br>30	0/30<br>1/32<br>0	0<br>3.1<br>0	--<br>-1.48 : 4.5<br>--	0/0<br>0/0<br>0/0	0<br>0<br>0	--<br>--<br>--	0/0<br>0/0<br>0/0	--<br>--<br>--	--<br>--<br>--
Sa. identicus	ZIKVRio-U1	14	4	0/4	0	--	0/0	0	--	0/0	--	--
Ae. terrens	ZIKVRio-S1	21	21	0	0	--	0/0	0	--	0/0	--	-
Ae. scapularis	ZIKVRio-S1	14	42	1/42	2.3	-1.1 : 3.4	0/1	0	--	0/0	--	--
**Intrathoracic injection**			**Number injected**									
Hg. leucoceleanus	ZIKVRio-S1	10	32	--	--	--	32/32	100	100	10/32	31.2	1.0 : 8.9
Sa. albiprivus	ZIKVRio-S1	10	32	--	--	--	16/32	50	3.8 : 117.9	3/9	33.3[d]	--
Sa. identicus	ZIKVRio-S1	10	31	--	--	--	28/31	90.3	75.4 : 101.3	6/19	31.5[d]	--
Ae. terrens	ZIKVRio-S1	10	45	--	--	--	6/45	13.3	0.9 : 22.4	0/6	0	--
Ae. scapularis	ZIKVRio-S1	10	82	--	--	--	10/82	12.1	0.03: 12.9	0/10	0	--

a: infection rate (IR) was refers the proportion of mosquitoes with infected body (abdomen + thorax) among engorged individuals after an incubation period (dpi); *b:* dissemination rate (DIR) corresponds to the proportion of mosquitoes with infected head among mosquitoes with infected body (abdomen + thorax positive) whether orally challenged or intrathoracic injected; *c:*transmission rate (TR) represents the proportion of mosquitoes with infectious saliva among mosquitoes with disseminated infection; *d*: rate determined with a single experiment; *e*: Confidence Interval (95%).
